# Positive B cell flow cytometry crossmatch without detectable donor-specific antibodies: true or false reactivity?

**DOI:** 10.3389/fimmu.2025.1690461

**Published:** 2026-01-23

**Authors:** Sonali N. de Chickera, Sabina Al Agbar, Arpit Sharma, David Beaune, Eva A. Sidahmed, Lakshman Gunaratnam, Abubaker Sidahmed

**Affiliations:** 1 Department of Medicine, Division of Nephrology, McGill University Health Centre, Montreal, QC, Canada; 2 Department of Pathology and Laboratory Medicine, Schulich School of Medicine and Dentistry, Western University, London, ON, Canada; 3 Division of Nephrology, Department of Medicine, Schulich School of Medicine and Dentistry, Western University, London, ON, Canada; 4 Department of Pathology and Laboratory Medicine, London Health Science Centre, London, ON, Canada

**Keywords:** flow cytometry crossmatch, HLA antibody, kidney transplant, solid-phase assay, surrogate crossmatch

## Abstract

In kidney transplant workups, supplementing flow cytometry crossmatches (FCXMs) with solid-phase assays (SPAs) helps differentiate between true positive from false positive results, which can prevent unnecessary waitlist delays. This study presents an analysis of three discordant cases characterized by positive B-cell FCXM results and the absence of detectable donor-specific antibodies (DSAs). Peripheral blood samples were obtained from both recipients and donors for HLA typing, FCXM, antibody screening, and surrogate crossmatches, with expanded single antigen bead assays at One Lambda laboratory to detect unidentifiable antibodies. Initial FCXM positivity did not correlate with SPA-confirmed DSAs. However, surrogate crossmatch results varied: Recipient 1 had unacceptable antigen leading to a halted transplant, Recipient 2’s negative results allowed the transplant to proceed, and Recipient 3’s mixed results led to the decision not to proceed with the transplant due to an unacceptable antigen. The findings suggest that a positive FCXM, in the absence DSAs, may be interpreted as false positive, underscoring the necessity for comprehensive testing in the pre-transplant evaluation process. Employing multiple diagnostic techniques ensures more accurate risk assessments, improves transplant outcomes, and expands the pool of suitable donors, emphasizing the critical role of thorough HLA and non-HLA antibody evaluations.

## 1 Introduction

Kidney transplantation is the preferred method of renal replacement therapy for patients with end-stage renal disease, providing a significantly better quality of life compared to dialysis. Overall, transplantation is associated with nearly 50% lower risk of death than remaining on dialysis (1). A major barrier to transplantation is finding a suitable, matched donor for each potential recipient, which is critical for optimizing graft longevity and minimizing the risk of rejection in the new host.

The Human leukocyte antigen (HLA) system is crucial in organ transplantation, determining the compatibility between donors and recipients (2). Antibody-mediated rejection (AMR) is a primary cause of graft loss in kidney transplantation, particularly when there is antigenic mismatch between donor and recipient (3). HLA antibodies are identified as significant risk factors for various forms of allograft rejection, including hyperacute, acute, and chronic allograft rejections (2). Notably, pre-formed donor-specific antibodies (DSAs) are associated with a higher risk of early acute AMR and poorer graft outcomes, even in cases with a negative flow cytometry crossmatch (FCXM) (4–6). Therefore, detecting pre-existing HLA antibodies in potential kidney recipients is essential for accurate pre-transplantation risk assessment.

Detecting DSAs in a recipient’s serum against a specific donor allograft is typically achieved through a combination of cell-based assays and solid phase assays (SPAs). Among cell-based methods, the FCXM is the most sensitive for detecting DSAs (7). Although this technique does not distinguish between complement- and non-complement-binding antibodies, its prognostic value in pre-transplant screening is well established (8). In contrast, SPAs, such as Luminex^®^, offer heightened sensitivity for detecting lower titer antibodies, enabling precise identification of specific HLA antibodies with allele specificity. Complimenting cell-based FCXM with SPAs can enhance our ability to differentiate immunologically relevant positive FCXMs from false positives (9). This combined approach is crucial for increasing the number of safe, compatible kidney transplants. However, challenges persist in interpreting both cell-based methods and SPAs (10–13).

Non-HLA antibodies are increasingly recognized as key mediators of both acute and chronic kidney allograft injury. Targeting antigens such as angiotensin II type 1 receptor (AT1R), endothelin A receptor (ETAR), and MHC class I–related chain A (MICA), these antibodies disrupt endothelial integrity and promote inflammation. While HLA antibodies remain central to transplant immunopathology, non-HLA antibodies may act independently or synergistically with HLA antibodies to amplify immune-mediated graft damage and contribute to poorer long-term outcomes.

We present three clinically distinct cases of positive B cell FCXM in the absence of DSAs to illustrate the value of comprehensive workup strategies like Expanded Single Antigen Beads and Surrogate Crossmatches and to propose a unifying hypothesis for interpreting discordant results.

This report presents three complex living donor kidney transplant cases in which standard anti-HLA antibody screening failed to explain unexpected FCXM results, prompting the use of expanded testing strategies, including surrogate crossmatches and non-HLA antibody assays.

### 1.1 Case 1

A 67-year-old Caucasian male (blood type AB+) with a history of autosomal dominant polycystic kidney disease (ADPKD), hypertension, dyslipidemia, and gout initially presented with graft dysfunction more than a decade after his first transplant. He was referred for evaluation for a second transplant after returning to hemodialysis in 2019. His creatinine level one week post-initial transplant was 89 μmol/L. At the time of referral for re-transplantation, the patient had no detectable circulating HLA antibodies and a calculated panel reactive antibody (cPRA) score of 0%. Screening with multiple sera and extensive troubleshooting yielded persistently negative HLA antibody results, despite a positive B cell FCXM with a new living donor. Non-HLA antibody tests, including AT1R and ETAR, were also negative. However, high-resolution donor typing revealed substantial haplotype overlap with the initial donor, and surrogate crossmatches identified specific class II antigens associated with reactivity.

### 1.2 Case 2

A 57-year-old Caucasian male (blood type A+) with ADPKD, hypertension, polycystic liver disease, and an inguinal hernia presented for pre-emptive transplantation evaluation. He had no history of sensitizing events, and his routine HLA antibody screening was repeatedly negative. Despite the absence of DSAs, B cell FCXM with his potential living donor (spouse) returned positive results. The patient had stable renal function on pre-transplant labs, and Non-HLA antibody tests, including AT1R and ETAR, were tested. Expanded bead-based testing revealed low-level class I and class II antibodies not detected on the standard panel, raising questions about clinical significance. Surrogate crossmatches for these targets were negative.

### 1.3 Case 3

A 15-year-old Caucasian male (blood type A+) with renal coloboma syndrome, bilateral renal dysplasia, and stage V CKD was assessed for his first kidney transplant. His medical history was notable for morbid obesity (BMI >35), growth hormone deficiency, asthma, and kyphosis. He had no prior dialysis or sensitizing exposures. Laboratory screening revealed a PRA of 0% and negative DSAs, yet FCXM with his donor (a family friend) was unexpectedly positive for B cells. Expanded testing identified a weak class I antibody that did not match donor HLA alleles, and a low-intensity DR17 antibody that yielded indeterminate results upon repeat testing. Mixed surrogate crossmatch outcomes further complicated the clinical decision. Non-HLA antibody tests, including AT1R and ETAR were tested.

## 2 Materials and methods

All testing was performed at the Histocompatibility and Immunogenetics Laboratory, London Health Sciences Centre, University Hospital Campus (London, Ontario, Canada) unless otherwise specified.

### 2.1 Sample storage and collection

Peripheral blood was collected from three potential kidney transplant recipients and their respective living donors (**Table 1**). Freshly separated donor lymphocytes were used for FCXM. Recipient sera were aliquoted and stored at -20°C for antibody analysis. DNA for HLA typing was isolated and stored at -80°C.

**Table 1:** Recipient and donor demographics.

Subject	Blood group	Sex	Type of donor	Age (years)	Ethnicity	Primary renal disease	Dialysis
Recipient 1	AB+	Male		67	Caucasian	ADPKD	Yes
Donor 1	O+	Male	Living Unrelated	46	Caucasian		
Donor 2	O+	Female	Living Unrelated	67	Caucasian		
Recipient 2	A+	Male		57	Caucasian	ADPKD	Pre-emptive
Donor 1	O+	Female	Living Unrelated	50	Caucasian		
Recipient 3	A+	Male		15	Caucasian	Renal colobama syndrome	Pre-emptive
Donor 1	A+	Female	Living Unrelated	47	Caucasian		

### 2.2 Flow cytometric crossmatch

Both IgG and IgM allo-FCXMs were conducted prospectively using donor peripheral blood T and B-lymphocytes. In each tube, at least 500,000 pronase-treated donor cells were incubated with 50 uL of recipient serum for 30 minutes. Following incubation, cells were washed and then labeled with anti-CD3 conjugated with PE and anit-CD19 conjugated with PC5 (BD Biosciences, San Jose, CA, USA) then incubated for an additional 15 minutes in the dark. Subsequent wash steps were performed before secondary antibody staining with F(ab)2 anti-human IgG conjugated with FITC (Jackson ImmunoResearch, West Grove, PA). Following a final 15-minute dark incubation and wash, samples were analyzed using a FACScan instrument (Beckman coulter). Interpretation was based on the right “shift” in mean channel fluorescence. At our institution, a positive reaction is defined as an increase in median channel shift (MCS) of fluorescence that is two standard deviations above the negative control for both T cell and B cells, on a 1024 scale. A surrogate FCXM methodology involves mixing recipient serum with lymphocytes from a third-party individual who shares the specific HLA alleles of the intended donor. This process detects whether the recipient has antibodies that target these donor antigens, which would lead to a positive and incompatible crossmatch result, potentially causing organ rejection.

### 2.3 Auto-flow cytometric crossmatch

Auto-FCXM was performed using the same protocol described above (Section 3.2), with recipient lymphocytes incubated with the recipient’s own serum.

### 2.4 HLA typing

Low-resolution HLA typing was conducted using polymerase chain reaction (PCR) with sequence-specific oligonucleotide probes (One Lambda). For high-resolution typing, supplementary PCR with Sequence-Specific Primers (PCR-SSP) was used to assess HLA-A, B, C, DRB1-5, DQA, DQB1, DPA1 and DPB1 (One Lambda).

### 2.5 HLA antibody detection and luminex-based troubleshooting

Our institution follows the Halifax protocol, described here (14). Serum samples regularly collected from active transplant-listed recipients were used for HLA antibody screening. HLA antibody screening was conducted using Luminex^®^-based solid-phase assays. LABScreenTM PRA Class I and II and Single Antigen Beads (SAB) kits (One Lambda, Canoga Park, CA) were used to detect HLA class I (HLA-A, -B, and -Cw) and class II (HLA-DR, -DQ, and -DP) antibodies. Fluorescence readings were obtained using the Luminex^®^ 200 analyzer and interpreted using HLA Fusion software (version 3.2, One Lambda). The calculated PRA (cPRA) was determined by summing individual antibodies with MFI values >1000 (15).

To resolve potential false negatives and mitigate assay interference (e.g., prozone effect), multiple troubleshooting steps were applied:

Serum Dilution: Aliquots of non-heat-treated recipient sera were tested at 1:10 and 1:50 dilutions in Luminex^®^ wash buffer (One Lambda).

Heat Inactivation: Sera were incubated at 56°C for 30 minutes in a water bath for heat inactivation, then centrifuged at 13,000 rpm for 20 minutes to remove supernatant debris.

Ethylenediaminetetraacetic acid (EDTA) Pre-treatment: 4 µL of a 0.1625M EDTA working solution was added to 105 µL of serum. Samples were then washed with wash buffer, and lipid supernatants were discarded.

Dithiothreitol (DTT) Pre-treatment: 5 µL of 0.005M DTT was added to sera and incubated at 37°C for 30 minutes to remove any IgM antibodies.

AdsorbOut™ (One Lambda) was used to reduce nonspecific background fluorescence.

Expanded antibody screening included LABScreen™ ExPlex Class I and II panels to detect additional HLA alleles not covered by standard kits.

### 2.6 Non-HLA antibody testing

Non-HLA antibody profiling was performed using LABScreen Autoantibody Group 1, Group 2, and Group 3 kits (One Lambda). Additionally, enzyme-linked immunosorbent assay (ELISA) kits were used to detect anti-AT1R and anti-endothelin type A receptor (ETAR) antibodies following the manufacturers’ protocol (One Lambda).

## 3 Results

### 3.1 Recipient 1: surrogate XM uncovers sensitization in repeat transplant candidate

A 67-year-old AB+ male with ADPKD was referred for a second kidney transplant. He previously received a living unrelated transplant in 2006 from his brother-in-law, which failed in 2017 due to chronic AMR, despite the absence of detectable DSAs at the time. The HLA typing revealed a 10/12 mismatch at 6 loci with the donor (**Table 2**). At the time, HLA typing standards were less comprehensive, and testing for HLA DQA1, DPA1, and DP alleles was not routinely performed across all centers due to their deemed limited clinical relevance. The patient returned to dialysis in 2019 and was re-listed for transplant with a cPRA of 0%.

**Table 2:** HLA typing of recipients and their respective donors.

Subject	Haplotype	A	B	C	DRB1	DRB 3/4/5	DQB1	DQA1	DPB1:	DPA1
Recipient 1	a	01:01	08:01	07:01	3:01	03*01:01	02:01	05:01	04:01P	01:03
	c	02:01	49:01	07:01	11:01	03*02:02	03:01	05:05	04:01P	01:03
Donor 1 (Brother-in-law)		03:01	07:02	07:02	15:01	05*01:01	06:02	01:01	04:01P	01:03
		11:01	14:02	08:02	01:02		05:01	01:02	02:01P	01:03
Donor 2 (Spouse)		03:01	07:02	07:02	15:01	05*01:01	06:02	01:01	03:01P	01:03
		02:01	35:01	04:01	01:03		05:01	01:02	04:01P	01:03
Recipient 2	a	01:01	57:01	06:02	7:01	4*03:01N	03:03	02:01	04:01P	01:03
	c	01:01	57:01	06:02	7:01	4*03:01N	03:03	02:01	13:01P	02:01
Donor 1 (Spouse)		01:01	07:02	07:01	3:01	03*01:01	02:01	01:02	04:01P	01:03
		29:02	08:01	07:02	15:01	05*01:01	06:02	05:01	05:01P	02:06
Recipient 3	a	01:01	08:01	07:01	01:01		05:04	01:02	02:01P	01:03
	c	23:01	49:01	07:01	09:01	04*01:03	03:03	03:02	03:01P	01:03
Donor 1 (Friend)		01:01	07:02	07:01	03:01	03*01:01	02:01	05:01	02:01P	01:03
		02:01	08:01	07:02	11:04	03*02:02	03:01	05:01	02:01P	01:03

The patient’s wife, the sister of the first donor, was identified as a potential living donor. FCXM with donor cells showed a positive B cell result with negative T cells. Multiple SAB assays, including troubleshooting with DTT, EDTA, serial dilution, heat inactivation, and Adsorb Out™, failed to reveal any HLA antibodies. One Lambda technical support conducted the standard LABScreen SAB assay and an expanded ExPlex SAB assay, which includes an additional 54 class I and 24 class II antigens beyond the standard assay (**Table 3**). Non-HLA antibodies, AT1R and ETAR, were negative (**Table 4**). However, surrogate crossmatches with DR15 and DQ6-positive cells were repeatedly positive (**Table 5**), prompting reclassification of those antigens as unacceptable. This increased the recipient’s cPRA from 0% to 42%.

**Table 3:** One Lamba single antigen bead and ExPlex class I and class II HLA antibody results.

Recipient ID	SAB I (MFI)	ExPlex class I (MFI)	SAB II (MFI)	ExPlex class II (MFI)
1	Negative	Negative	DQB1*06:02 (953)	Negative
2	B*15:12 (783)	C*07:01 (4198)	DRB4*01:01 (1688)	DPB1*105:01 (5028)
		C*12:02 (662)	DQB1*06:02 (1560)	DPB1*31:01 (1978)
		A*26:02 (642)	DRB1*08:01 (1474)	DRB1*14:04 (1183)
			DPB1*04:01 (1267)	DRB1*04:04 (978)
			DQB1*03:02 (1241)	
			DRB1*14:54 (1125)	
			DRB1*14:01 (1051)	
			DPB1*01:01 (1031)	
3	B*73:01 (398)	A*02:18/A*02:07 (1200)	DRB1*03:01 (1616)	DRB1*14:03 (553)
			DRB5*01:01 (784)	
			DRB1*03:02 (656)	
			DRB1*13:03 (612)	
			DPB1*01:01 (461)	
			DRB1*14:02 (392)	

Cells bolded and highlighted in grey denote DSAs as detected by the One Lambda laboratory.

**Table 4:** One Lambda non-HLA antibody results using the LABScreen assay.

Subject	Group 1	Group 2	Group 3
Recipient 1	Vimentin Protein kinase C, zeta Heterogeneous Nuclear Ribonucleoprotein K Regenerating Family Member 3 Alpha	N/A	Collagen III
Recipient 2	Glyceraldehyde 3-phosphate dehydrogenase Fibronectin Leucine Rich Transmembrane Protein 2 Chromatin Assembly Factor 1 Subunit B Agrin Protein kinase C eta type	N/A	Collagen III
Recipient 3	Fibronectin Leucine Rich Transmembrane Protein 2 Protein Tyrosine Phosphatase Receptor Type N Heterogeneous Nuclear Ribonucleoprotein K Regenerating Family Member 3 Alpha	N/A	Collagen III

**Table 5:** Recipient 1: Evaluating the clinical significance of DR15 and DQ6 through surrogate crossmatches in pre-transplant assessment.

Donor	A	B	C	DR	DQB	DQa	BW4	BW6	MESF NHS T cells	MESF T cells	ΔMESF	MESF NHS B cells	MESF B cells	Δ MESF B cells
											T cells			
1	2, 24	51, 57	6, 16	13, 15	6, -	1, -	pos		1259	961	**-299**	1684	3743	**2060**
2	2, -	7, 35	10, 7	4, 11	7, 8	3, 5	pos		1502	1511	**9.3**	1936	2033	**97**
3	1, 2	8, 49	7, -	17, 11	2, 7	5, -	Pos	pos	1792	1310	**-482**	3202	1732	**-1470**
4	1, 24	13,62	9, 6	4, 7	8, 9	2, 3	pos	pos	1096	986	**-110**	1472	1126	**-346**
5	3, 24	65, 35	4, 8	1, -	5, -	1, -	pos	pos	1381	1353	**-28**	1728	1353	**-375**
6	3, 24	7, 62	9,7	13, 15	6, -	1, -	pos	pos	1260	1148	**-113**	2144	4341	**2197**
7	2,3	7,35	4,7	01:03, 15	5,6	1, -		pos	1765	1638	**-127**	1605	5345	**3739**
8	2, 24	8, 18	5, 7	17, 13	2, 6	1, 5	pos	pos	1345	1141	**-204**	2269	1452	**-817**
9	1, 3	7, 8	7,-	11, 15	7, 6	1, 5		pos	1755	1360	**-395**	1611	3406	**1794**
10	24, 68	44, -	5, -	13, 16	5, 6	1,-	pos		1794	1528	**-266**	4246	1835	**-2411**
11	2, 26	64,44	8,16	17, 7	2, -	2, 5	pos	pos	1465	1229	-236	1981	1434	-546
12	1, -	8, -	7, -	17, -	2, -	5, -		pos	1198	972	-226	1457	972	-485
13	24, 68	65, 48	4, 8	4, 13	7, 8	3, 5	pos	pos	1554	1452	-102	1728	1280	-448
14	11, 34	18, 51	12, 14	7,15	2, 5	1, 2	pos	pos	1608	1530	-77	1560	4808	3248
15	1,3	7,37	6,7	15,-	6,-	5,-	pos	pos	1226	1,074	-152	2045	8315	6270
16	24,29	13,62	1,6	1,12	5,7	1,5	pos	pos	1328	1,244	-85	2648	1865	-783
17	24,34	7,81	7,8	15,17	2,6	1,5	pos	pos	1169	983	-187	2128	6359	4231
18	1,1	7,8	5,7	17,4	2,7	3,5		pos	1177	1,004	-173	1431	1048	-383
19	2,-	7,51	1,4	1,15	5,6	1,-	pos	pos	1712	1,398	-314	1610	3911	2300
20	29,31	44,51	15,16	4,7	2,8	2,3	Pos		1986	1,391	-594	2643	1525	-1118
21	1,3	35,51	7,14	12,15	5,7	1,5	Pos	Pos	930	837	-93	1571	8734	7163
22	3,68	18,44	9,7	11,12	7,-	5,-	Pos	Pos	1127	1,235	109	1474	1028	-446
23	2,-	60,44	10,5	1,9	9,5	1,3	Pos	Pos	1189	938	-251	1638	1095	-543
24	2,68	13,35	4,6	1,11	7,5	1,5	pos	pos	1123	1019	-104	1607	1255	-352

In the Δ MESF B cells column, green cells signify a negative surrogate crossmatch, whereas red cells denote positive surrogate crossmatches. Borderline result (Donor 9) is highlighted in yellow.

Bold values showed Delta MESF for T- and B- cell crossmatch.

Outcome: Transplant was declined based on surrogate XM findings indicating previously unrecognized sensitization. However, the patient were transplanted later using deceased donor kidney with negative FCXM.

### 3.2 Recipient 2: low-level DSAs with negative surrogates and excellent graft function

A 57-year-old A+ male with ADPKD and no history of sensitizing events was assessed for a pre-emptive transplant, donated by his spouse. This recipient had an 11/12 mismatch with the donor (**Table 2**). Routine FCXM showed B cell positivity with negative T cells and negative auto-FCXM. Standard SAB testing revealed no HLA antibodies. The recipient’s cPRA is 0% and non-HLA antibodies, AT1R and ETAR, were negative.

Expanded SAB testing by One Lambda identified weak Class II DSAs (DQB1*06:02, DPB1*04:01) and a Class I antibody (C*07:01) (**Table 3**). Notably, the SAB panel lacked a specific C*07:01 bead, only detecting the closely related C*07:02. Surrogate FCXM against these antigen-expressing cells were negative (**Table 6**).

**Table 6:** Recipient 2: Evaluating the clinical significance of DP4 and DQ6 through surrogate crossmatches in pre-transplant assessment.

Donor	A	B	C	DR	DQ	DP	BW4	BW6	MESF NHS T cells	MESF T cells	ΔMESF T cells	MESF NHS B cells	MESF B cells	Δ MESF B cells
1	24,32	7,27	2,7	4,15	6,8	04:01,04:01	Bw4	Bw6	1232	1527	**295**	4291	5403	**1112**
2	2,2	18,51	1,7	1,11	5,7	03:01,04:01	Bw4	Bw6	1164	1088	**-76**	3167	2940	**-227**
3	24,24	27,35	4,15	4,11	3,4	02;01,04:01	Bw4	Bw6	1000	905	**-95**	2897	3039	**142**
4	2,11	7,7	7,7	15,15	6,6	04:01,20:01		Bw6	1140	1466	**326**	2460	4008	**1548**
5	2,2	7,8	7,7	1,17	2,5	01:01,03:01		Bw6	983	1394	**411**	2110	2918	**808**
6	1,29	7,45	6,7	13,15	7,6	04:01,04:01		Bw6	912	1059	**147**	3376	3145	**-231**
7	2,24	62,60	9,10	10,13	5,6	03:01,04:01	Bw4	Bw6	1049	596	**-453**	5527	2803	**-2724**
8	1,24	8,61	2,7	1,17	2,5	01:01,04:02	Bw4	Bw6	1064	1111	**47**	3168	2928	**-240**
9	3,24	7,44	7,7	7,15	2,6	26:01,-	Bw4	Bw6	915	853	**-62**	5188	3781	**-1407**
10	2,29	44,50	6,16	7,15	2,6	04:01,13:01	Bw4	Bw6	1184	1011	**-173**	4688	4352	**-336**
11	2,24	38,38	4,7	15,15	5,5	01:01,05:01	Bw4		1109	1053	**-56**	3622	2723	**-899**
12	2,30	39,41	7,7	8,13	7,6	02:01,02:01		Bw6	1236	1122	**-114**	2254	3502	**1248**
13	3,32	7,44	5,7	1,15	5,6	02:01,04:02	Bw4	Bw6	1571	1405	**-166**	3115	2439	**-676**
14	32,33	44,58	10,4	17,7	2,2	02:01,04:01	Bw4		1041	746	**-295**	2488	1511	**-977**

In the Δ MESF B cells column, green cells signify a negative surrogate crossmatch, whereas red cells denote positive surrogate crossmatches. Borderline result is highlighted in yellow.

Bold values showed Delta MESF for T- and B- cell crossmatch.

After multidisciplinary review, the transplant proceeded given the weak and likely clinically irrelevant antibody profile.

Outcome: Successful transplant with stable renal function at one year.

### 3.3 Recipient 3: indeterminate DR17 signal and mixed surrogate results lead to conservative decision

A 15-year-old A+ Caucasian male with renal coloboma syndrome and stage V chronic kidney disease (CKD) was evaluated for a living unrelated transplant (**Table 2**). Auto-FCXM was negative and B cell FCXM was positive. No HLA antibodies were detected on standard SAB testing, and cPRA was 0%. Non-HLA antibodies, AT1R and ETAR, were negative.

ExPlex identified a weak A2 antibody (A*02:18, 02:07) not matching the donor’s likely A*02:01, and a Class II DR17 antibody (MFI 1616). However, this signal was indeterminate on repeat testing (MFI 566) and did not appear on other bead platforms. Surrogate crossmatches using DR17-positive donors showed mixed results (2 positive, 4 negative - **Table 7**), raising uncertainty about true sensitization.

**Table 7:** Recipient 3: Evaluating the clinical significance of DR17 through surrogate crossmatches in pre-transplant assessment.

HLA typing									T cells			B cells		
Donor	A	B	C	DR	DQ	DP	BW4	BW6	MESF NHS T cells	MESF T cells	ΔMESF T cells	MESF NHS B cells	MESF B cells	Δ MESF B cells
1	3,25	39,49	7, 12	1,11	7,5	04:02P, 04:01P	4	6	825	805	**-20**	1767	1457	**-310**
2	11,24	13,15	9, 4	8,15	4,5	21:01, 02:02	4	6	843	665	**-178**	1502	1172	**-330**
3	2,11	62,60	10,7	15,-	5,-	05:01, 01:01	6	6	1113	1244	**131**	1863	2058	**195**
4	1,3	8,35	4, 7	1,17	2,5	15:01, 01:01	6	6	1293	1086	**-207**	1739	1337	**-402**
5	3,11	62,35	4, 12	7,15	9,6	04:01, 03:01	6	6	1217	942	**-275**	4953	1369	**-3584**
6	3,32	7,64	7, 8	7,-	2,9	13:01, 02:01	4	6	511	451	**-60**	2823	1602	**-1221**
7	24,31	71,60	10,-	17,11	2,7	04:01, 01:01	04	6	1356	719	**-637**	1924	2250	**326**
8	2,29	44,44	7,16	11,7	2,7	04:01,-	4	4	1176	1373	**197**	3767	1421	**-2346**
9	1,2	8,40(60)	3(10),7	17,-	2	04:01P, 01:01P		present	914	988	**74**	3951	39090	**35139**
10	1,2	60,52	10,12	1,7	2,5	03:01P, 04:01P	present	present	925	1031	**106**	4192	2925	**-1267**
11	3,23	7	7	8,15	4,6	04:01P,	present	present	1227	1005	**-222**	5460	4091	**-1369**
12	1,24	27,49	2,7	11,13	7,6	02:01P, 04:01P	present		1166	970	**-196**	3221	2410	**-811**
13	3,68	7	7	103,15	7,6	04:01P, 04:02P		present	1371	1300	**-71**	2696	2206	**-490**
14	3,30	7,51	7,16	13,15	6	04:01P	present	present	1103	937	**-166**	3694	2465	**-1229**
15	1,3	7,60	10,7	11,13	7,6	02:01P, 05:01P	present		1264	1004	**-260**	4414	2805	**-1609**
16	1,2	7,8	7,-	17,11	2,7	02:01P,-	6		1400	1498	**98**	1849	17500	**15651**
17	1,29	8,44	7,16	17,7	2,-	19:01P, 04:02P	4	6	864	800	**-64**	1199	1900	**701**
18	30,32	8,55	5,7	17,7	2,-	14:01, 02:01	4	6	864	830	**-34**	1199	4000	**2801**

Each donor carried the DR17 antigen. In the Δ MESF B cells column, green cells signify a negative surrogate crossmatch, whereas red cells denote positive surrogate crossmatches. Borderline results are highlighted in yellow.

Showed Delta MESF for T- and B- cell crossmatch. It should be all bold.

Given the inconsistency and potential risk of undetected reactivity, the transplant was deferred.

Outcome: Transplant postponed out of caution due to inconclusive DR17 findings.

## 4 Discussion

Positive B cell FCXM results, in the absence of detectable DSAs, pose a critical interpretive challenge in pre-transplant immunological risk assessment. These discordant findings complicate clinical decision-making, particularly when transplant urgency or limited donor availability are factors. Our three-case series underscores the need for a layered diagnostic strategy that extends beyond conventional screening to uncover underlying sensitization or rule out spurious results. The variability in surrogate crossmatch outcomes among the three recipients highlights the complexity of these immunological profiles, illustrating that a positive FCXM without corroborating DSA evidence does not uniformly predict adverse transplant outcomes. Specifically, the halted transplant in Recipient 1, the successful transplant in Recipient 2, and the deferred transplant in Recipient 3 following mixed surrogate crossmatch results demonstrate the spectrum of clinical scenarios that may arise. The integration of solid-phase assays particularly expanded single antigen bead assays into the pre-transplant workup provides a higher resolution assessment of the antibody repertoire. This enable the detection of low-titer or non-HLA antibodies that may not be evident through conventional FCXM (16). As such comprehensive antibody profiling may reveal the presence of acceptable HLA mismatches (17), leading to more informed decisions regarding donor selection and risk stratification.

The aforementioned cases, illustrate how nuanced interpretation and expanded testing are essential to distinguishing true sensitization from false positivity, ultimately influencing clinical decision-making and outcomes. It is well established that both pre-existing and *de novo* HLA antibodies pose risks for rejection (18–20). Therefore, identifying DSAs during the pre-transplantation period is critical to avoiding donor-recipient pairings that lead to hyperacute or acute AMR (2, 21). As demonstrated by the cases, A key challenge, lies in the interpretation of a positive FCXM when no HLA antibodies are detectable. The risk of proceeding with transplantation under these circumstances is less certain. Careful consideration is required when deciding on further workup, especially for recipients who have been on the waitlist for several years or have limited donor options. To address this, we used comprehensive troubleshooting, expanded HLA testing, non-HLA antibody testing, and surrogate FCXMs to accurately assess any significant clinical risks for each recipient. Specifically, discordant B cell FCXMs necessitate layered testing to accurately assess clinical relevance.

The FCXM is the gold standard cell-based method of HLA antibody detection because it allows independent assessment of T and B cells, demonstrating clinically relevant antibodies and determining compatibility between donor and recipient (7). Labelling T cell using anti-CD3 and B cell using anti-CD19 allow this technique to include HLA class I and II markers to look for variability of class I and II expressions on different donor populations. However, FCXMs may produce false-positive results for several reasons: non-specific binding to donor lymphocytes (auto-FCXM positivity), incomplete donor HLA typing (e.g., missing DQA, DPA, or other loci, which can lead to undetected antibodies against untyped antigens or alleles), and alloantibodies reacting with shared epitopes (22). Furthermore, FCXM also lacks specificity for non-HLA antibodies. As such, FCXM results should be interpreted alongside molecular typing methods and SPAs in pre-transplant risk assessments. While multiplex SAB assays are the most common and sensitive methods for detecting HLA antibodies, interpreting these tests can present challenges. For example, technical variation such as serum and reagent degradation can affect results, leading to clinically irrelevant to false positive signals. The strengths of FCXM’s, such as its high sensitivity and ability to detect complement-fixing antibodies, are counterbalanced by limitations in specificity, potentially leading to false-positive results that complicate the pre-transplant evaluation (23–27).

Despite their sensitivity, SAB assays can yield false negatives due to the ‘prozone effect,’ where IgM antibodies block IgG binding to beads or complement components interfere (7, 28). To improve IgG binding, patient sera can be incubated with DTT to denature IgM antibodies (29). Additionally, complement activity can be mitigated by using EDTA in the wash buffer (23, 30, 31). Schnaidt et al. (24) demonstrated that pre-treating serum with standard heat inactivation can also eliminate the prozone effect, revealing potentially significant HLA antibodies. Furthermore, strongly binding HLA antibodies may only become detectable upon serum dilution in SAB testing. Non-specific serum factors can also adsorb to latex beads in SPAs, causing high background fluorescence that masks true positives. AdsorbOut™, which consists of microparticles in a blocking solution without specific antigen coating, can reduce this background interference (25). Importantly, Rituximab, which eliminates B-cells before antigen exposure and disrupts their differentiation into antibody-secreting cells, can lead to false-positive B cell FCXMs (26, 27). However, in the 3 cases presented here, none of the recipients received Rituximab.

There is no universally accepted ‘cut-off’ MFI for positivity in SAB testing, leading each laboratory to set its own threshold based on clinical experience. Our center uses a cut-off MFI of >1000 to determine the significance of HLA antibodies in kidney transplant recipients. Additionally, variations in laboratory techniques and machine calibrations can lead to discrepancies in HLA antibody results, as evidenced by differences between One Lambda and our laboratory’s SAB results. In our practice generally we consider MFI of 500–999 as indeterminant (weak) and anything below MFI of 500 is negative. We use the Baseline Normalized value which is the raw MFI data minus the Background raw MFI of the Negative serum. Non-HLA antibodies, such as those targeting AT1R, have been implicated in graft dysfunction and rejection, particularly through endothelial injury (32). However, AT1R is primarily expressed on vascular endothelial and smooth muscle cells, with limited or absent expression on resting B or T lymphocytes i.e might not affect FCXM results. Although some studies suggest that activated lymphocytes may transiently express components of the renin-angiotensin system, this expression is minimal and unlikely to produce significant interference in FCXM assays (32, 33). We assessed our recipients’ serum for non-HLA antibodies, including AT1R and anti-ETAR, using One Lambda multiplex assays and ELISAs. Although we detected some non-HLA antibodies, their clinical relevance remains unclear in our cases. In our cases, all AT1R and ETAR testing was negative, and no consistent patterns emerged between non-HLA antibody profiles and FCXM positivity. Thus, while non-HLA antibody testing can add value in certain transplant contexts, it is unlikely to explain the isolated positive B cell FCXM results observed in our cohort and may be less relevant to the central question addressed in this paper.

We regularly treated our cells with pronase (0.5‐1 mg/mL) to mitigate false‐positive results in B‐cell FCXMs, which can occur due to nonspecific immunoglobulin binding by Fc receptors and the binding of surface immunoglobulins onto B‐cells. Pronase treatment is known to reduce high background antibody binding often associated with B-cell FCXMs by incubating target lymphocytes with pronase. Despite these precautions, there is no history of rituximab treatment in these patients, and the use of pronase did not alter the outcomes of their tests. These strategies highlight the complexities in interpreting pre-transplant immunological risk and underscore the need for a comprehensive approach to improve risk stratification and patient outcomes. Comprehensive antibody testing, including both HLA and non-HLA antibodies, is crucial for evaluating transplant candidates.

After extensive evaluation of each recipient-donor pair, our transplant team made decisions based on clinical risk and other pertinent factors. For recipient 1, who had previously received a kidney transplant, we discovered that the recipient’s second donor (wife) shared a haplotype with the first donor. Despite the absence of identifiable DSAs through testing and troubleshooting, we identified DRB1*15:01 and DQB1*06:02 as unacceptable antigens through surrogate crossmatches with other donor cells. Consequently, the recipient’s cPRA was changed from 0% to 42%, and the transplant did not proceed. Alternative options for this recipient include joining the kidney paired donation list, finding another living donor, or staying on the deceased donor list. This case highlights the value of surrogate XM in identifying unacceptable mismatches. This real risk for transplantation is only revealed using surrogate XM and thorough investigation to reveal first donor and second donor are related. Recipient 2, found to have a class I DSA and two low-level class II DSAs by One Lambda testing (**Table 3**), had no prior sensitizing events, making the clinical significance of these findings uncertain. However, subsequent surrogate crossmatches against these antibodies were negative, allowing the transplant to proceed. The patient’s graft function remained stable for one-year post-transplantation. This case shows that not all weak DSAs are clinically significant. For recipient 3, despite two positive and four negative surrogate crossmatch results, DR17 was not considered a DSA (**Table 7**). Although DR17 tested negative, precautions should still be taken. A donor with DR17 should be avoided to prevent the potential for a retrospective positive, which could compromise the transplant outcome. Ultimately, our lab deemed DR17 was unacceptable, and the recipient has not proceeded to transplant.

Finally, while non-HLA antibodies were tested for, definitive conclusions or associations are not able to be made with these results, as their implications remain unclear.

While our findings reinforce current understanding of the challenges in interpreting positive B cell FCXM without detectable DSAs, these cases add important real-world nuance. Each case illustrates a distinct scenario in which discordant FCXM and SAB results required individualized interpretation. Our experience highlights the limitations of relying solely on standard SAB testing or FCXM results in isolation. In particular, the underrepresentation of certain alleles (e.g., DR17) in SAB panels, variability in surrogate crossmatch results, and lack of clear clustering patterns in phenotype beads complicate risk stratification.

Collectively, our findings support a stepwise diagnostic framework that integrates standard and expanded SAB testing, surrogate crossmatches, and clinical context to guide transplant decision-making. This layered approach can reduce the risk of inappropriate donor exclusion while safeguarding against underrecognized immunologic incompatibility. In all these three cases we performed expanded beads, non-HLA testing, various treatment for prozone effects, and surrogate crossmatches, although the most test was the surrogate crossmatch we do not have to discourage the comprehensive approach, specifically the treatment to mitigate assay interference i.e. the presence of prozone effect.

## 5 Conclusions

To conclude, a positive FCXM with a negative HLA antibody screen might initially suggest a ‘false positive’. However, it is crucial for laboratories to thoroughly rule out undetectable DSAs using advanced screening techniques beyond routine tests. Failing to detect HLA antibodies due to false-negative results can lead to poor clinical outcomes, such as inappropriate donor-recipient matching, AMR, and an increased need for immunosuppression. Conversely, excessively cautious interpretations may keep patients on dialysis longer, increase mortality risks, and potentially overlook viable opportunities for successful transplantation. Therefore, employing comprehensive methods to detect HLA antibodies is essential throughout the pre-transplant assessment process. These cases underline that single-test protocols may be inadequate, and careful interpretation of HLA antibody results is vital to enhance the clinical benefits of transplantation. Our experience emphasizes the advantage of working up recipients for allografts from living unrelated donors, which allows time for thorough investigation. Although using deceased donors might necessitate a simplified protocol due to time constraints, such thorough evaluations are crucial for optimal outcomes.

## Data Availability

The raw data supporting the conclusions of this article will be made available by the authors, without undue reservation.

## References

[1] Kaballo MA Canney M O’Kelly P Williams Y O’Seaghdha C . A comparative analysis of survival of patients on dialysis and after kidney transplantation. Clin Kidney J. (2018) 11:389–93. doi: 10.1093/ckj/sfx117, PMID: 29942504 PMC6007575

[2] Lefaucheur C Loupy A Hill GS Andrade J Nochy D Antoine C . Preexisting donor-specific HLA antibodies predict outcome in kidney transplantation. J Am Soc Nephrol. (2010) 21:1398–406. doi: 10.1681/ASN.2009101065, PMID: 20634297 PMC2938596

[3] Parajuli S Aziz F Garg N Panzer SE Joachim E Muth B . Histopathological characteristics and causes of kidney graft failure in the current era of immunosuppression. World J Transplant. (2019) 9:123–33. doi: 10.5500/wjt.v9.i6.123, PMID: 31750089 PMC6851501

[4] Picascia A Infante T Napoli C . Luminex and antibody detection in kidney transplantation. Clin Exp Nephrol. (2012) 16:373–81. doi: 10.1007/s10157-012-0635-1, PMID: 22552384

[5] Haarberg KM Tambur AR . Detection of donor-specific antibodies in kidney transplantation. Br Med Bull. (2014) 110:23–34. doi: 10.1093/bmb/ldu005, PMID: 24736012

[6] Pape L Becker J Immenschuh S Ahlenstiel T . Acute and chronic antibody-mediated rejection in pediatric kidney transplantation. Pediatr Nephrol. (2015) 30:417–24. doi: 10.1007/s00467-014-2851-2, PMID: 24865478

[7] Alheim M Paul PK Hauzenberger DM Wikström AC . Improved flow cytometry based cytotoxicity and binding assay for clinical antibody HLA crossmatching. Hum Immunol. (2015) 76:849–57. doi: 10.1016/j.humimm.2015.09.047, PMID: 26429307

[8] Guillaume N . Improved flow cytometry crossmatching in kidney transplantation. HLA. (2018) 92:375–83. doi: 10.1111/tan.13403, PMID: 30270577

[9] Bray R Nickerson P Kerman R Gebel H . Evolution of HLA antibody detection: technology emulating biology. Immunol Res. (2004) 29:41–54. doi: 10.1385/IR:29:1-3:041, PMID: 15181269

[10] Pei R Wang G Tarsitani C Rojo S Chen T Takemura S . Simultaneous HLA Class I and Class II antibodies screening with flow cytometry. Hum Immunol. (1998) 59:313–22. doi: 10.1016/S0198-8859(98)00020-2, PMID: 9619770

[11] Patel AM Pancoska C Mulgaonkar S Weng FL . Renal transplantation in patients with pre-transplant donor-specific antibodies and negative flow cytometry crossmatches. Am J Transplant. (2007) 7:2371–7. doi: 10.1111/j.1600-6143.2007.01944.x, PMID: 17845571

[12] Tait BD Hudson F Brewin G Cantwell L Holdsworth R . Solid phase HLA antibody detection technology--challenges in interpretation. Tissue Antigens. (2010) 76:87–95. doi: 10.1111/j.1399-0039.2010.01486.x, PMID: 20403141

[13] Bettinotti MP Zachary AA Leffell MS . Clinically relevant interpretation of solid phase assays for HLA antibody. Curr Opin Organ Transplant. (2016) 21:453–8. doi: 10.1097/MOT.0000000000000326, PMID: 27200498 PMC4936436

[14] Liwski R Greenshields A Conrad D Murphey C Bray R Neumann J . Rapid optimized flow cytometric crossmatch (FCXM) assays: The Halifax and Halifaster protocols. Hum Immunol. (2018) 79:28–38. doi: 10.1016/j.humimm.2017.10.020, PMID: 29109009

[15] Wahrmann M Hlavin G Fischer G Marinova L Schwaiger E Hörl W . Modified solid-phase alloantibody detection for improved crossmatch prediction. Hum Immunol. (2013) 74:32–40. doi: 10.1016/j.humimm.2012.10.012, PMID: 23073293

[16] Schinstock C Stegall MD . Acute antibody-mediated rejection in renal transplantation: current clinical management. Curr Transplant Rep. (2014) 1:78–85. doi: 10.1007/s40472-014-0012-y, PMID: 27656351 PMC5027607

[17] Ichinohe T Uchiyama T Shimazaki C Matsuo K Tamaki S Hino M . Feasibility of HLA-haploidentical hematopoietic stem cell transplantation between noninherited maternal antigen (NIMA)-mismatched family members linked with long-term fetomaternal microchimerism. Blood. (2004) 104:3821–8. doi: 10.1182/blood-2004-03-1212, PMID: 15280193

[18] Heilman R Nijim A Desmarteau Y Khamash H Pando MJ Smith M . De novo donor-specific human leukocyte antigen antibodies early after kidney transplantation. Transplantation. (2014) 98:1310–5. doi: 10.1097/TP.0000000000000216, PMID: 24879384

[19] Kanter Berga J Pallardo Mateu LM Beltran Catalan S Puig Alcaraz N Sancho Calabuig A Gavela Martinez E . Donor-specific HLA antibodies: risk factors and outcomes after kidney transplantation. Transplant Proc. (2011) 43:2154–6. doi: 10.1016/j.transproceed.2011.06.053, PMID: 21839219

[20] Willicombe M Brookes P Sergeant R Santos-Nunez E Steggar C Galliford J . De novo DQ donor-specific antibodies are associated with a significant risk of antibody-mediated rejection and transplant glomerulopathy. Transplantation. (2012) 94:172–7. doi: 10.1097/TP.0b013e3182543950, PMID: 22735711

[21] Howell WM Harmer A Briggs D Dyer P Fuggle SV Martin S . British Society for Histocompatibility & Immunogenetics and British Transplantation Society guidelines for the detection and characterisation of clinically relevant antibodies in allotransplantation. Int J Immunogenet. (2010) 37:435–7. doi: 10.1111/j.1744-313X.2010.00955.x, PMID: 20670336

[22] Stefoni S Nanni-Costa A Buscaroli Borgnino A Iannelli S Raimondi C Scolari MP . Validity of flow cytometry for cross-match evaluation in clinical renal transplantation. Nephron. (1991) 57:268–72. doi: 10.1159/000186274, PMID: 2017265

[23] Visentin J Vigata M Daburon S Contin-Bordes C Fremeaux-Bacchi V Dromer C . Deciphering complement interference in anti-human leukocyte antigen antibody detection with flow beads assays. Transplantation. (2014) 98:625–31. doi: 10.1097/TP.0000000000000315, PMID: 25058376

[24] Schnaidt M Weinstock C Jurisic M Schmid-Horch B Ender A Wernet D . HLA antibody specification using single-antigen beads--a technical solution for the prozone effect. Transplantation. (2011) 92:510–5. doi: 10.1097/TP.0b013e31822872dd, PMID: 21869744

[25] Zerrouki A Ouadghiri S Benseffaj N Razine R Essakalli M . High background in Luminex^®^ assay for HLA antibody screening: Interest of Adsorb Out™. Transpl Immunol. (2016) 36:20–4. doi: 10.1016/j.trim.2016.03.001, PMID: 27004694

[26] Desoutter J Apithy MJ Bartczak S Guillaume N . False positive B-cells crossmatch after prior rituximab exposure of the kidney donor. Case Rep Transplant. (2016) 2016:4534898. doi: 10.1155/2016/4534898, PMID: 27239362 PMC4864553

[27] Reindl-Schwaighofer R Oberbauer R . False-positive CDC x-match after rituximab. Transpl Int. (2014) 27:e124–5. doi: 10.1111/tri.12385, PMID: 24964286

[28] Vaidya S Cooper TY Avandsalehi J Barnes T Brooks K Hymel P . Improved flow cytometric detection of HLA alloantibodies using pronase: potential implications in renal transplantation. Transplantation. (2001) 71:422–8. doi: 10.1097/00007890-200102150-00015, PMID: 11233905

[29] Carey SB Boswijk K Mabrok M Rowe P Connor A Saif I . A reliable method for avoiding false negative results with Luminex single antigen beads; evidence of the prozone effect. Transpl Immunol. (2016) 37:23–7. doi: 10.1016/j.trim.2016.04.002, PMID: 27109036

[30] Guidicelli G Anies G Bachelet T Dubois V Moreau JF Merville P . The complement interference phenomenon as a cause for sharp fluctuations of serum anti-HLA antibody strength in kidney transplant patients. Transpl Immunol. (2013) 29:17–21. doi: 10.1016/j.trim.2013.09.005, PMID: 24056164

[31] Schwaiger E Wahrmann M Bond G Eskandary F Böhmig G . Complement component C3 activation: the leading cause of the prozone phenomenon affecting HLA antibody detection on single-antigen beads. Transplantation. (2014) 97:1279–85. doi: 10.1097/01.TP.0000441091.47464.c6, PMID: 24621535

[32] Reindl-Schwaighofer R Heinzel A Gualdoni GA Mesnard L Claas F Oberbauer R . Novel insights into non-HLA alloimmunity in kidney transplantation. Transpl Int. (2020) 33:5–17. doi: 10.1111/tri.13546, PMID: 31650645 PMC6972536

[33] Sorohan BM Ismail G Leca N Tacu D Obrișcă B Constantinescu I . Angiotensin II type 1 receptor antibodies in kidney transplantation: An evidence-based comprehensive review. Transplant Rev (Orlando). (2020) 34:100573. doi: 10.1016/j.trre.2020.100573, PMID: 33002671

